# Chirurgus on Cancer of the Jaws

**Published:** 1831-01-01

**Authors:** 


### XXXVIII.

### CHIRURGUS ON CANCER OF THE JAWS.

I take up the pen to add my mite to the cases of "cancer of the jaws," which you have published in your last number, (October, 1830) and to endeavour "to decide if M. Delpech is right or wrong in his opinions respecting their origin." That the mucous membrane of the nares, including that which lines the various cells communicating with them, is subject to nearly all the variety of tumours, from the delicate vesicular polypus to the medullary sarcoma, is verified by the different authorities on the subject, from Astruc's Traité des Tumeurs, to Travers on Malignant Diseases; or by an examination of one or two musea of pathological anatomy. Now, if we find the same vesicular polypus growing, with its delicate pedicle, from the mucous membrane investing the parietes of the antrum maxillare, that we do from the membrane clothing the ethmoid bone, is it at all surprising we should find the same vascular sarcoma in the antrum that we do in the nares, or even in the œsophagus? Is it in the least degree marvellous that we should find the same medullary sarcoma, or milt-like tumour in the antrum that we do in the stomach? They grow from the same mucous membrane, the gastro-pulmonary. "Scirrhous cancer," says Mr. Travers, "originates in some or other description of secretory structure—its seats are the follicles of the mucous membrane." "Medullary cancer affects any or all textures, without exception." "Cancer of the antrum begins upon the lining membrane." "Cancerous fungus of the antrum grows from the whole surface of the cell. I have seen it (says Mr. Travers,) commencing in and proper to the nares—it is a growth to which the common lining membrane is subject." "Polypi, vesicular and fleshy, form in all the cells and chambers of the bones of the face and head". Besides the many dissections recorded of tumours in the antrum, the most satisfactory of which are to be found in the "Memoires de l'Academie Royale de Chirurgie," I have now examined six, but could find no disease affecting any of the neighbouring nerves; they were only involved in the general mass. I should, therefore, presume that "the posterior palatine nerves, and the spheno-palatine ganglion with its filaments, as far as the superior maxillary nerve" mentioned by Delpech, were excited to assume the same morbid action—"the medullary disease"—exactly in the same manner as the nerves of a stump, after amputation, have their neurilematic sheaths thickened with lymph, and become involved in the bony deposit which sometimes takes place from the edges of the sawn bones.

There are some cases lately published in the medical periodicals to which I beg to call your attention, and one of them, I observe has the morbid appearances described after death. It is detailed in the Lancet for the 10th April, 1830, but no mention is made of any disease having attacked the nerves, although the tumour extended upwards, and involved the brain itself. Similar cases are mentioned in Hodgkin's Catalogue of Preparations in the Museum of Guy's Hospital, wherein one of them began in the sphenoidal cells. In the same periodical, another case is described, wherein the entire upper jawbone was excised, by Mr. Lizars, in the infirmary of this place, and which I had the good fortune to witness. I examined carefully the morbid growth after its removal, but could not perceive any diseased nerves exterior to the bony parietes. I have also, since reading M. Delpech's views, examined the pathological collections of this city, but could not discover any diseased nerves in any specimen. I saw Mr. Liston remove the upper jaw-bone the other day in the infirmary for diseased antrum, but could not observe any disease of the palatine nerve. Excision of the whole upper jaw-bone, which I have now seen done by our two great surgeons, Liston and Lizars, is a more scientific operation, and a more certain cure for these diseases of the antrum, than that followed by M. Delpech, which we, in our younger days, witnessed with horror, besides being attended with failure. In March last, I visited a man labouring under an acute form of disease of the antrum maxillare, and, to my surprise, he completely recovered.

His face was enormously swollen, the skin erysipelatous, the eye of the affected side shut up, the mouth with difficulty admitted of being opened half an inch—a profuse discharge of ichorous matter flowed from the antrum, at an opening above the bicuspides and at one of their alveolar sockets, the teeth of the affected side having fallen out. The palatine plate, the alveolar processes, and the outer wall of the antrum, were all soft, spongy, and tumefied. On incising both of these points with the bistoury, matter flowed, and on inserting a probe, it passed deeply into the antrum, apparently among thick, pulpy substance.

Incisions were repeated, every second or third day, for fifteen or twenty days, when the tumefaction, discharge of matter, and all remains of suppuration and inflammation, disappeared. On first view of this patient, I considered him past all relief. I presumed that the antrum contained a tumour of the medullary character, and that it had advanced so as to excite erysipelas, with hectic or irritative fever.

The medullary tumour which attacks the gum of the upper jaw, near the bicuspides, I have seen mistaken for the protrusion of the medullary sarcoma from the antrum.

Yours,

Chirurgus.

